# Direct Delivery of Antigens to Dendritic Cells via Antibodies Specific for Endocytic Receptors as a Promising Strategy for Future Therapies

**DOI:** 10.3390/vaccines4020008

**Published:** 2016-03-28

**Authors:** Christian H. K. Lehmann, Lukas Heger, Gordon F. Heidkamp, Anna Baranska, Jennifer J. Lühr, Alana Hoffmann, Diana Dudziak

**Affiliations:** 1 Laboratory of DC-Biology, Department of Dermatology, Friedrich-Alexander-Universität Erlangen-Nürnberg, University Hospital Erlangen, Hartmannstr. 14, Erlangen 91052, Germany; christian.lehmann@uk-erlangen.de (C.H.K.L.); lukas.heger@uk-erlangen.de (L.H.); gordon.heidkamp@uk-erlangen.de (G.F.H.); baranska@ciml.univ-mrs.fr (A.B.); jennifer.lühr@uk-erlangen.de (J.J.L.); alana.hoffmann@uk-erlangen.de (A.H.); 2 Centre d’Immunologie de Marseille-Luminy, Aix Marseille Université, INSERM-CNRS, Marseille-Luminy 13288, France

**Keywords:** antigen targeting, antigen targeting antibodies, cancer, CLR, DC, DCIR, DEC205, dendritic cell subsets, moDC, vaccine

## Abstract

Dendritic cells (DCs) are the most potent professional antigen presenting cells and are therefore indispensable for the control of immunity. The technique of antibody mediated antigen targeting to DC subsets has been the basis of intense research for more than a decade. Many murine studies have utilized this approach of antigen delivery to various kinds of endocytic receptors of DCs both *in vitro* and *in vivo*. Today, it is widely accepted that different DC subsets are important for the induction of select immune responses. Nevertheless, many questions still remain to be answered, such as the actual influence of the targeted receptor on the initiation of the immune response to the delivered antigen. Further efforts to better understand the induction of antigen-specific immune responses will support the transfer of this knowledge into novel treatment strategies for human diseases. In this review, we will discuss the state-of-the-art aspects of the basic principles of antibody mediated antigen targeting approaches. A table will also provide a broad overview of the latest studies using antigen targeting including addressed DC subset, targeted receptors, outcome, and applied coupling techniques.

## 1. Introduction

One of the crucial abilities of the immune system is the distinction between self- and pathogen-derived antigens. Professional antigen presenting cells, especially Dendritic cells (DCs), not only present engulfed and processed self- and foreign antigens as peptide MHC complexes (pMHC) on their surface, they also reflect their environment by the surface expression status of co-stimulatory molecules (e.g., CD80 (B7-1), CD86 (B7-2)), activation markers (CD40, CD83), and the secretion level of cytokines (e.g., IL-12) and chemokines [1,2]. Thus, the presentation of pathogen-derived peptides in an inflammatory context allows for the induction of protective immune responses against the invading pathogen, while the presentation of (self-)antigens in a non-inflammatory context maintains peripheral tolerance [2,3,4,5,6,7,8,9,10,11,12,13]. These capabilities define DCs as one of the key players within the immune system.

## 2. Pattern Recognition Receptors

Germline-encoded pattern recognition receptors (PRRs) enable DCs to detect danger signals such as conserved pathogen-associated molecular patterns (PAMPs) or other shared structures of various pathogens like fungi, bacteria, helminthes, and viruses (reviewed in [10,14,15,16,17,18,19,20,21,22]). Triggering of PRRs, which are also expressed on a variety of other immune cells, can lead to the uptake of pathogens or pathogen-derived material and/or to cell activation [12,23,24]. Besides nucleotide-binding oligomerization domain proteins (NOD) or retinoic acid inducible gene 1-like receptors (RIG-I), two prominent PRR family members are Toll like receptors (TLRs) and C type lectin receptors (CLRs, Clec) [22].

TLRs are type I integral membrane glycoproteins and well characterized members of the PRR family. They are localized in the plasma membrane (TLRs 1, 2, 4, 5, 6, 11) or in endosomal compartments (TLRs 3, 7, 8, 9) [14,25,26]. The endosomal TLRs are responsible for sensing nucleic acids such as bacterial or viral RNAs and DNAs, while the TLRs of the plasma membrane are able to recognize pathogen-derived sugars, lipoproteins, protozoa, or fungal cell wall components. TLR ligand recognition leads to signal transduction, transcription factor activation, and finally DC maturation including upregulation of co-stimulatory molecules and secretion of pro-inflammatory cytokines [22,27]. The recognition of PAMPs is guided by leucine-rich repeats, while the cytoplasmic Toll/IL-1 receptor (TIR) domain is responsible for downstream signaling [15].

CLRs are a diverse family of calcium dependent molecules situated in the cellular plasma-membrane. They can be distinguished into type I (DEC205, MMR) and type II transmembrane proteins (almost all other CLRs), displaying the N-terminus either outside or inside the cell, respectively. By their carbohydrate recognition domain (CRD), CLRs are able to sense self- and non-self-sugar side chains of proteins such as N-glycans, O-glycans, and glycosphingolipid motifs [28]. The specificity for mannose enables the detection of viruses, fungi, and mycobacteria, while the specificity for fucose allows for the recognition of certain bacteria and helminths. Moreover, glucan structures are expressed by mycobacteria and fungi [29,30]. CLRs are capable to not only bind the sugar side chains; they also trigger endocytosis of the bound material. This process can then lead to processing and presentation of antigens as peptide-MHC complexes on the DC surface. In dependency of the intracellular signaling motif, which can be an inhibitory ITIM (immunoreceptor tyrosine-based inhibitory motif) or activating ITAM (immunoreceptor tyrosine-based activating motif), binding of a natural ligand to the CLR can induce either inhibiting or activating signaling pathways in the cell, respectively. The latter ones (in combination with TLR signaling or outside type I interferon) are important for a full DC maturation and presentation of antigens under inflammatory conditions [14,26,29].

Besides the recognition of pathogens, DCs are also capable to sense, take up, process, and present self-antigens derived from apoptotic cells, cell debris, or damaged cells, which are recognized as damage–associated molecular patterns (DAMPs) [31,32,33]. This detection and uptake is implemented by the expression of scavenger receptors as well as CLRs, as shown for Clec9A [34]. Due to defective ribosomal products (DRIPs), which are derived from short lived, aborted, or mis-folded proteins, also self-peptides can be presented on the DC surface [35]. The presentation of self-peptides is necessary to counterbalance potential auto-reactive T cells, which might have escaped negative selection processes in the thymus. T cells, which interact with self-peptides complexed to MHC molecules on immature/semi-mature DCs under steady-state conditions undergo anergic mechanisms (unresponsiveness) or will be deleted. Therefore, antigen presentation by steady-state DCs is an important checkpoint for the maintenance of peripheral tolerance. This control might be accompanied by the help of regulatory T cells [5,6,7,36,37].

Overall, the ability of effective antigen uptake, processing, and presentation as well as the regulation of immunogenic and tolerogenic immune responses renders DCs as promising candidate cells for immunotherapeutic approaches [38,39,40,41,42,43].

## 3. Monocyte-Derived DCs in Immunotherapeutic Approaches

**Figure 1:**
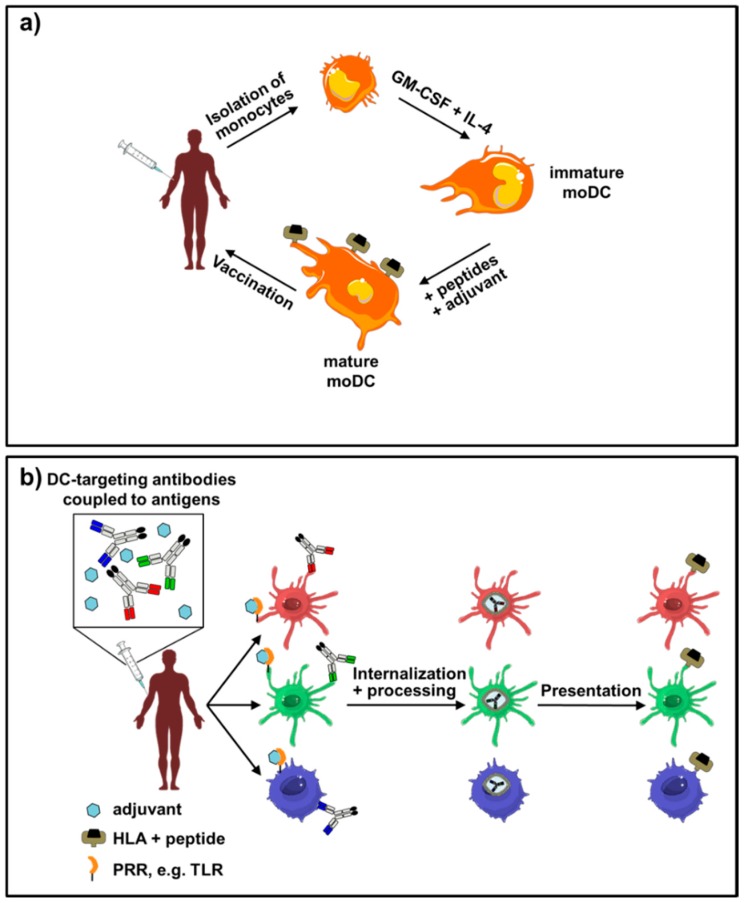
Principles of the use of human dendritic cells (DCs) for the treatment of diseases. There are two principal approaches to use DCs for the treatment of patients either by (**a**) using monocyte-derived DCs (moDCs) or by (**b**) directly targeting DCs in the patient using DC-targeting antibodies coupled to antigens. (**a**) For the vaccination of patients with their own moDCs, monocytes are isolated from the blood of the patient and differentiated into moDCs by culturing them in media containing GM-CSF and IL-4 for 5–6 days. Subsequently, cells are loaded with antigens and either matured with adjuvants (e.g., cytokine cocktail consisting of IL-1β, IL-6, TNFα, and PGE_2_) or kept immature. These cells presenting peptides of the antigen as peptide-MHC complexes on their surface are then transfused back into the patient to induce either an inflammatory T cell response (matured DCs) or tolerance (immature DCs); (**b**) in an alternative approach, antigens are targeted directly to DCs *in vivo* by fusion of the antigen to antibodies directed against DC surface molecules. After binding to the DCs, the antibodies are internalized, processed, and presented on MHC class I and II molecules on the DC surface. Analogous to moDCs, the DCs induce an inflammatory T cell response in the presence of adjuvants such as toll like receptor (TLR) ligands or tolerance, if the antibody is injected alone without adjuvant. By usage of antibodies directed against surface molecules selectively expressed on one DC subset (here differentially colored in red, green, and blue), the type of immune response can be further regulated due to different functions of the DC subsets. Templates from Servier Medical Art (www.servier.com) were used and adapted for this figure.

As DCs are essential for the presentation of antigenic peptides to T cells and thereby enabling them to elicit potent antigen-specific immune responses to pathogens and tumor cells, the idea of utilizing DCs for cancer treatment has already emerged several years ago [43,44]. One fundamental initial discovery for current therapeutic approaches was that human peripheral blood monocytes could be differentiated into monocyte-derived DCs (moDCs) by a combination of growth factors and cytokines, namely GM-CSF (granulocyte macrophage colony-stimulating factor) and IL-4 (Interleukin 4) (Figure 1a) [45,46,47,48,49].

*In vitro* differentiated moDCs share many similarities with primary DCs found in human peripheral blood, indicated by their potential to activate and differentiate naïve T cells into effector T cells [50]. This is especially effective, when they are matured with single maturation stimuli (e.g., αCD40 antibody, TLR ligands, including LPS, pIC, or CpG) or maturation cocktails (IL-1β, PGE_2_, IL-6, TNFα) [45,46,48,49,51,52,53]. In recent years, moDCs have been generated for self-vaccination of otherwise incurable tumor patients [54]. Importantly, the production of therapeutic moDCs needs to be conducted under good manufacturing practice (GMP) conditions, including their differentiation from blood monocytes. These cells are then loaded with antigenic peptides [42,51,54,55,56,57], soluble proteins [58], or tumor lysates [40,54,59,60,61,62,63,64,65,66], or by the transfection of tumor epitope-encoding mRNAs [54,67,68,69,70,71,72,73], DNAs [74,75,76,77], or whole tumor mRNA [40,54,78], accompanied by protocols ensuring a full DC maturation (Figure 1a). This maturation process seems to be a critical step in the production of therapeutic moDCs, as the appropriate time point and the maturation cocktail composition determine the efficiency of the peptide-loaded moDCs to migrate into the patients’ lymph nodes [41,43,49,79,80,81]. Several studies have been initiated using moDCs in the treatment of (mostly) stage-4 melanoma, prostate, pancreatic, and breast cancer, as well as glioblastoma, where a significantly prolonged overall survival of those patients could be documented [42,49,57,58,59,62,63,68,81,82,83,84,85,86,87,88]. Although moDC-based therapies increased the life expectancy of certain types of formerly incurable cancer patients, the response rate is still lower than desired [38,54,56,57,59,64,68,80,81,86,88,89,90,91,92]. Of great interest, treatment with checkpoint inhibitors in combination with antigen-loaded moDCs might further increase the overall survival rate. Future clinical studies will be indispensable to clarify the efficacy of this new combinatorial therapeutic approach [93,94,95].

## 4. Delivery of Antigens to DC Subsets by Usage of Recombinant Antibodies

Besides therapeutic approaches utilizing moDCs, other approaches in tumor vaccination strategies have been considered in human trials and preclinical models [96], such as immunization with tumor peptides [97,98,99,100,101,102], tumor-derived DNA [103,104], glycan-modified tumor antigens [105], liposomes [106,107,108], or even by injection of whole tumor lysates [109,110,111]. As described before, antigens can be provided to DCs by various techniques such as RNA or DNA electroporation, injection of soluble proteins, nanoparticles, liposomes, or long peptides. However, not all of these techniques can be used to specifically address antigens to DCs directly *in vivo*. Also the delivery of antigenic peptides by loading DCs with long peptides is not DC or antigen-presenting cell (APC) specific and bears the risk of the unintentional induction of tolerance to pathogen-derived antigens [112]. The usage of undirected nanoparticles or liposomes most likely target highly phagocytically active macrophages rather than DCs. Therefore, a more DC-specific antigen delivery technique is required. In consideration of those less specific antigen delivery techniques, the idea arose that a selective delivery of antigens to antigen presenting cells *in vivo* would be favorable for a better immune response against the targeted antigen without unwanted spreading of antigens, the need of cell isolation, cell manipulation, or *in vitro* moDC generation [9,43,44]. The last three points seem to be of critical importance, as DCs are very sensitive to experimental manipulations demonstrated by immediate changes in the DC activation status and phenotype in culture systems, thus no longer reflecting their natural *in vivo* phenotype [2,4,113]. Moreover, the newest findings suggest that moDCs should be rather allocated to the family of monocytes than to DCs [50]. Especially, this last issue might be important to understand the difficulties observed with moDC-based therapies, as monocytes themselves are less efficient than DCs in the activation of T cells upon peptide MHC (pMHC) complex presentation [114]. Thus, a directed delivery of antigens to the APCs in the most appropriate tissue might harbor the possibility of a better specificity on the one hand, but also a broader therapeutic application as needed for the treatment of infectious diseases, cancer, and autoimmune diseases on the other hand.

A precise delivery of antigens into DCs *in vivo* requires knowledge on the expression of endocytic receptors and the targeted DC subset. Today, mainly two strategies for a specific transport of antigens to DCs *in vivo* are discussed. Both take advantage of the specific binding of an antibody to an endocytic receptor on the DC surface, ensuring the delivery of the cargo into the antigen-processing machinery (Figure 1b). However, these two approaches differ in the entities coupled to these antibodies. One possibility is to couple them to nanoparticles or to liposomes containing the antigen of choice, which has been thoroughly reviewed elsewhere [115,116,117]. The second strategy is also known as antibody mediated antigen targeting and will be discussed in more detail in this review (Figure 1b, Table 1). Besides its endocytic capacity, the chosen receptor should only be expressed on professional APCs, such as DCs. Otherwise the distribution of the antigen among many different cells might lead to tolerogenic reactions, e.g., the induction of regulatory T cells. For this purpose, endocytic receptors naturally detecting pathogens, such as C type lectin receptors, have emerged as promising targeting receptors.

Once an endocytic candidate receptor has been selected and antibodies have been generated and tested, the antigen of choice can be coupled to these specific antibodies. This coupling process can either be accomplished by chemical linking of the antibody to the antigen to be delivered, or by production of a recombinant antibody-antigen fusion construct. For the production of these recombinant fusion constructs, the variable regions of the parental antibody are cloned by conventional methods or by using a phage display library. There are several advantages in using recombinant antigen-coupled antibodies in comparison to chemically conjugated antibodies. One important fact is the knowledge of the exact number of antigen molecules coupled. Second, it is more feasible to control the endotoxin concentrations by using an antibody-antigen cloning strategy. Another major advance is the possibility to genetically modify the targeting antibodies. In this way it is possible to not only optimize the production of the antibody itself, but to also modify its host compatibility (by humanization), its solubility (by changing amino acid composition), its fixation of complement components (by changing the glycosylation sites), its binding to Fc receptors (by changing glycosylation or using Fab or scF_v_s (single chain F_v_s)), and finally also its dimerization tendencies [118,119,120,121,122,123,124,125]. These facts allow for commercial GMP-compatible upscaling of the production of an antigen targeting antibody. Another research tool might be to utilize biotinylated antibodies together with Streptavidin-coupled antigens as recently demonstrated [126].

## 5. Antigen Targeting Receptors

### 5.1. DC Subsets

Several distinct DC subsets have been described in lymphoid and non-lymphoid organs of mouse and man. Depending on their localization, their cell surface marker and transcription factor expression, as well as their migratory potential, DCs can be roughly distinguished into plasmacytoid DCs (pDCs) and conventional (classical) DCs. The latter can be further separated into resident and migratory DCs [11,50,127]. In mice, pDCs express low to absent levels of CD11c and MHC class II, but high levels of B220, Bst-2, and Siglec-H. Interestingly, CCR9 (CDw199) has become a marker for the distinction of CCR9 negative mature and CCR9 positive immature pDCs [128,129]. pDCs are able to produce high amounts of type I interferon upon virus encounter or TLR9 stimulation [130,131,132,133,134,135,136]. Despite this strong ability to boost the initiation of an immune response, the role of pDCs in antigen presentation is under current debate [21,137,138,139,140,141,142,143,144,145,146].

In contrast to pDCs, murine conventional DCs are prone to present antigens to naïve CD4^+^ and CD8^+^ T cells. Resident conventional DCs are localized in lymphoid organs such as lymph nodes, spleen, or Peyer’s patches and can be distinguished by the expression of CD11c and MHC class II in combination with CD8, CD11b, CD172a (SIRPα), and XCR1 into CD11c^+^MHC-II^hi^CD11b^-^CD8^+^XCR1^+^ (further referred to as resident CD8^+^ DCs) and CD11c^+^MHC-II^hi^CD11b^+^CD8^−^SIRPα^+^ DCs (further referred to as resident CD8^−^ DCs) [3,147,148]. Non-lymphoid migratory DCs are localized in non-lymphoid tissues and can be distinguished by the expression of CD103, CD207, and CD11b, which is also true for skin DCs [11,127,149]. They are highly positive for MHC class II and are––especially after antigen uptake and maturation––able to migrate from their respective peripheral tissues into lymphoid tissues, preferentially into draining lymph nodes (e.g., dermal dendritic cells [148,150]).

In humans, DCs can be distinguished into CD1c^+^ DCs, CD141^+^ DCs, and pDCs. The functional specialization of human DC subsets is under great debate and current knowledge has been reviewed elsewhere [151,152,153,154,155,156].

### 5.2. Antigen Targeting to DCs and DC Subsets by Utilizing C Type Lectin Receptors

The identification of endocytic receptors specifically expressed by certain DC subsets of mice and the generation of recombinant murine DC subset-specific antigen targeting antibodies made it possible to target antigens to single DC subsets in mice *in vivo* [3,8,157,158,159,160]. In this section, we focused on several important examples. However, due to space constraints we were unable to discuss all receptors and all studies using a specific receptor and have therefore included a table with a more comprehensive list of citations (Table 1).

The first proof-of-principle was provided by utilizing a specific C type lectin receptor antibody for DEC205 (Ly-75, DEC-205, CD205). DEC205 is harboring 10 carbohydrate recognition domains and has a molecular weight of 205 kDa [161]. This receptor was shown to be highly expressed on resident CD8^+^ DCs and thymic epithelial cells [148,153,161]. Some studies also propose a weak expression on B cells, T cells, and granulocytes [114,162,163,164]. Importantly, upon antigen encounter, mimicked by the usage of chimeric receptors, it has been demonstrated that DEC205 internalizes and recycles very efficiently via MHC-II positive cellular compartments back to the surface [165]. The natural ligand of DEC205 remains still to be found, but some studies suggest apoptotic and necrotic material as well as CpG motifs as consecutive ligands [166,167,168]. A recent study also suggested the plasminogen activator Pla of *Yersinia pestis* as a potential receptor ligand responsible for the bacterial dissemination [169]. Another C type lectin receptor, the Dendritic Cell Inhibitory Receptor 2 (DCIR2, antibody clone 33D1, Clec4A, CD367), is exclusively expressed by resident CD8^−^ splenic DCs in mice [3,170,171,172]. In humans, a relative of DCIR2 (DCIR) can be found on all blood DC subsets, monocytes, and granulocytes [170,173,174]. The natural ligand for DCIR has not yet been found, however there are studies suggesting a possible role in recognizing HIV glycoproteins [175,176,177]. Upon antibody binding to human DCIR, Meyer-Wentrup, *et al.* demonstrated that DCIR is internalized into endosomal/lysosomal compartments and was able to induce T cell responses [143].

As the receptors DEC205 and DCIR2 have often been used in comparative studies, we summarized these results in the following section. By *in vivo* antigen targeting of murine resident CD8^−^ DCs in comparison to targeting of CD8^+^ DCs with antigen-carrying DCIR2 (33D1) or DEC205 antibodies, it became possible to preferentially induce either CD4^+^ or CD8^+^ T cell responses *in vivo*, respectively [3]. These experiments indicated that targeting CD8^+^ DCs is an efficient way to induce cross-presentation, whereas targeting of CD8^−^ DCs is superior for processing and presentation of antigens through the traditional MHC class II pathway. The findings further suggested that this was not due to the receptor that was targeted, but rather due to an intrinsic difference in the MHC presentation capacities of the DC subsets under steady-state conditions [3,7,8,157,200].

Subsequent studies demonstrated that antigen targeting via the DEC205 or the DCIR2 receptor does not only lead to an expansion of transferred antigen-specific T cells in the beginning, but also to their depletion after several days. This was indicated by experiments, in which low doses of antibodies were administrated in the absence of co-stimulatory stimuli leading to the induction of peripheral tolerance under the control of regulatory T cells. These regulatory responses were very efficient as mice were protected from type 1 diabetes, experimental autoimmune arthritis, and experimental autoimmune encephalomyelitis (EAE) [3,7,8,157,187,191,194,195,197,198,200,236].

It was also shown that DEC205 or DCIR2 antibodies can be utilized under immunostimulatory conditions to induce protective cellular and humoral responses *in vivo* needed for the fight against pathogens and malignancies [3,170,179,180,182]. This has, for example, been shown for *Yersenia pestis* [182], *Plasmodium yoelii* [180], dengue virus [171], HIV [183,237], the cancer/testis antigen 1A (known as CTAG1A or NY-ESO-1), or the Her2/neu breast cancer antigen in a protective model with NT2.5 tumor cells [189]. Interestingly, Neubert, *et al.* could demonstrate the induction of both protective and therapeutic anti-tumor responses in a murine melanoma model, which was independent of the initially targeted DC subset [179].

Furthermore, other receptors, mainly belonging to the C type lectin family, and their corresponding antibodies have been used for the induction of immune responses against various pathogens and cancer types *in vivo*. Here, we will present the most prominent studies in closer detail.

Human DC-specific intercellular adhesion molecule 3-grabbing non-integrin (DC-SIGN, Clec4L, CD209) has been demonstrated to recognize a variety of sugar residues including mannose residues on HIV virus particles. DC-SIGN is naturally expressed on moDCs, macrophages, and liver epithelial cells [238,239,240]. Importantly, a true homologue of human DC-SIGN has not been identified in mice yet, thus making it difficult to dissect functional aspects of DC-SIGN *in vivo*. The nearest homologues regarding ligand specificity are SIGN-R1 and SIGN-R3, which are both members of a family of eight different SIGN-Rs [241,242,243]. However, it has also become evident that the underlying signaling cascades differ in mice and humans [241]. To better understand, whether DC-SIGN might be useful for the delivery of antigens in humans, an interesting mouse model was generated expressing the human DC-SIGN molecule under the control of the CD11c promoter [244]. Although utilizing the CD11c promoter, which is not selectively active in DCs, might not be the best choice, the authors found the uptake of antigens via the DC-SIGN receptor to mainly induce Th2 immunity and regulatory responses [29,245]. Others observed potent CD4^+^ and CD8^+^ T cell responses *in vivo* and *in vitro* [219,220,221,232]. In a primate model with *Macacca fascicularis* it could also be shown that antigen targeting antibodies directed against macaque DC-SIGN were uptaken into myeloid APCs and Kupffer cells of the liver [222].

The member 9A of the C type lectin family (Clec9A, DNGR-1, CD370) is highly expressed on conventional CD8^+^ or CD103^+^ DCs and their precursors, but also weakly on pDCs in mice. In humans, Clec9A expression was found on CD103^+^ intestinal and CD141^+^ DCs [209,211,246,247,248,249,250,251]. Studies using targeting antibodies coupled with the model antigen ovalbumin showed the generation of potent cytotoxic CD8^+^ T cell responses and the reduction of tumor load in an intravenous injection model of the pseudo-metastasing ovalbumin expressing melanoma cells [209]. Additionally, it has been demonstrated that this strategy is also suitable for eliciting CD4^+^ T cell as well as antibody responses [210,211,212]. Importantly, the phenotype of the differentiated T cells is completely dependent on the applied adjuvant and antibody dosage. In more detail, the injection under non-inflammatory conditions led to the generation of a regulatory T cell response. Interestingly, in dependency of the administered adjuvant, the co-injection with poly(I:C) induced Th1 responses, while co-administration of Curdlan elicited Th17 responses [210]. Another not further investigated finding is that targeting of Clec9A seems to be especially suitable for the differentiation of naïve CD4^+^ T cells into follicular T helper cells (Bcl6^+^CXCR5^+^PD-1^+^CH2D1A^+^IL-21^+^ICOS^+^) [212]. However, the mechanism of this process is not clear yet.

The macrophage mannose receptor (MMR, recently also MR, Clec13D, CD206) is not only expressed on macrophages, endothelial cells, and smooth muscle cells of the trachea, but also on mature and immature moDCs and on human peripheral blood CD1c^+^ DCs *in vivo* [206,252,253]. Studies taking advantage of MMR-specific antibodies coupled to tumor antigens, such as pmel17 (pre-melanosome protein 17, also known as gp100) and hCG-β (human chorionic gonadotropin β) induced an MHC-I and MHC-II presentation and an antigen-specific T cell proliferation [224,225,226]. Another study focused on the tumor antigen NY-ESO1, which was similarly coupled to an MMR antibody. Here, the authors demonstrated a pronounced CD8^+^ cytotoxic T cell response [206] suggesting that the chosen antigen might influence the resulting immune response.

The lectin-like oxidized low-density lipoproteine receptor 1 (LOX-1, Clec8A) is a receptor for heat-shock proteins (e.g., Hsp70 [230]) and apoptotic cellular fragments. It is an important player in immunological reactions to these proteins and apoptotic material and is also expressed on DCs [230,232]. The potency of LOX-1 was first demonstrated by targeting of the model antigen ovalbumin via a recombinant antibody to LOX-1. Interestingly, the authors found that the induced immune responses led to a rejection of the ectopically ovalbumin expressing lymphoma cell line E.G7 *in vivo* [230,232]. The analyses of antigen-specific CD4^+^ T cell responses revealed cytokine secretion of IL-2, IFNγ, and TNFα when the T cells were re-stimulated with antigen loaded bone marrow derived DCs (BMDCs) [230]. Recently, LOX-1 antigen targeting was approved for a study in rhesus macaques, in which the induction of influenza virus neutralizing antibodies was demonstrated after administration of hemagglutinin coupled LOX-1-specific targeting antibodies *in vivo* [231].

A further interesting targeting molecule is the ITAM-containing receptor Dectin-1, also known as Clec7A. Dectin-1 can bind β-glucans and is therefore important for the uptake of yeast and yeast-derived particles (Zymosan). The molecule is reported to be expressed on murine cDCs, Langerhans’ cells (LCs), and in lower amounts also on murine pDCs [17,18,254,255]. In humans Dectin-1 can be found on all monocyte populations as well as macrophages, DCs, neutrophils, B cells, and eosinophils [17,18,256]. Data demonstrated that in macrophages the endocytosis via Dectin-1 is independent of Syk activity, while ligand recognition in murine DCs triggers Syk and CARD9 activation [257]. This results in the transcription of innate immune response genes [258]. Notably, Dectin-1 can shuffle bound soluble proteins into the cell, while the receptor recycles back to the cell surface. An antibody specific for Dectin‑1 coupled to ovalbumin has been demonstrated to induce not only CD4^+^ and CD8^+^ T cell responses, but also ovalbumin-specific antibody responses [215]. The same group has further investigated the expression of Dectin-1 in more detail and found it present on CD11c^+^MHC-II^hi^CD11b^+^CD8^−^CD4^−^ DCs, which is, however, in contrast to previous reports showing expression also on CD11c^+^MHC-II^hi^CD11b^+^CD8^−^CD4^+^ DCs [259]. In contrast to murine studies, the delivery of the antigens hemagglutinin, flu-M1, or MART-1 to Dectin-1 on moDCs led to an activation of the targeted moDCs, the expansion of flu-M1 as well as MART-1-specific T cells, but also to a re-stimulation of memory Th17 cells. Besides CD4^+^ T cell responses, Dectin-1 targeting was also promoting the differentiation of naïve CD8^+^ T cells into flu-M1-specific cytotoxic CD8^+^ T cells [216,217].

Langerin (CD207 or Clec4K) is a key marker molecule of Langerhans’ cells. Langerin is not only found on epidermal Langerhans’ cells, but also on migratory Langerhans’ cells and some dermal DC subsets, as well as on migratory DCs in lymph nodes [260]. As the receptor is especially present on those cell subsets, it attracted scientists to study the function of those cell subsets in the initiation of immune responses, as Langerin is involved in antigen recognition and uptake. The expression of Langerin is also connected to the presence of Birbeck granules [261]. The targeting of antigens via targeting antibodies to Langerin-expressing cells, including Langerhans’ cells, but also CD103^+^CD207^+^ dermal DCs and CD8^+^ lymph node resident DCs, has been demonstrated to induce CD8^+^ and CD4^+^ T cell responses to ovalbumin and p24 of HIV after *in vivo* injection into naïve C57Bl/6 mice [178,184]. In contrast to this, Langerin targeting has been shown by Flacher, *et al.* to induce cross-tolerance in a B16F10-ova expressing tumor model, even when it is used *in vivo* with a strongly activating TLR ligand such as Imiquimod [218]. The same group also reported that ovalbumin targeting to Langerin was not inducing CD4^+^ or CD8^+^ T cell responses in contrast to DEC205 targeting to Langerhans’ cells [202]. Recently, also Idoyaga, *et al.* demonstrated Langerhans’ cells to induce tolerance without any co-stimulation to the MOG peptide in an EAE model [172], thereby suggesting a rather tolerogenic role after Langerin-specific antigen uptake.

Additionally, the direct targeting of antigens to pDCs using specifically expressed C type lectins has been evaluated by taking advantage of antibodies specific for Siglec-H, which is a highly endocytotically active receptor found on pDCs and pDC precursors [234]. Zhang, *et al.* also demonstrated Siglec-H targeting to be effective in the induction of naïve CD8^+^ T cell responses by combining a chemically coupled Siglec-H-ova conjugate and the TLR stimulus CpG [234]. By using a genetically coupled Siglec-H antibody without any co-stimulation Loschko, *et al.* demonstrated the potency of this method in inhibiting T cell dependent autoimmune reactions in an EAE model with MOG peptide as antigen [141].

Another promising and interesting receptor for antigen targeting is the C type lectin-like receptor DC asialoglycoprotein receptor (DC-ASGPR, Clec10A, MGL, CD301) mainly expressed on immature DCs and granulocytes, but not on monocytes [262]. Antigen targeting of hemagglutinin with recombinant antibodies to DC-ASGPR on IFNα-matured human moDCs did not only induce an antigen-specific CD4^+^ T cell proliferation, but also their predominant differentiation into IL-10 producers and to a lesser extend also IL-2 and IFNγ [232]. This was true for the coupling of auto-antigens, such as prostate specific antigen (PSA) as well as foreign antigens, e.g., hemagglutinin (HA) [232].

### 5.3. Other Receptors Used for Antigen Delivery to APCs

The strategy of using antigen coupled antibodies against specifically expressed surface endocytic receptors is not limited to C type lectin receptors. In fact, all receptors that fulfill important criteria, such as expression pattern, endocytic activity, routing to different compartments, as well as availability of suitable antibodies, can potentially be utilized. In the next section, we will provide some examples of other non C type lectin and non lectin-like receptors, which have been utilized in a range of studies. Due to space constraints we could not discuss all possible candidates, but rather tried to discuss the most common and interesting examples. Also the targeting via Fc receptors using immune complexes will not be part of this review, as this strategy is not specific for a certain target receptor, but rather for a group of receptors dictating the outcome of this technique.

MHC-II is the molecule responsible for the presentation of antigenic self and foreign peptides to CD4^+^ T cells. It is constitutively expressed at high levels on all professional antigen presenting cells, such as DCs and B cells. Castro, *et al.* utilized different antibodies for various receptors, e.g., CD11c, MHC-II, and DEC205 to compare their efficiencies in antigen targeting. They chemically coupled the model antigen ovalbumin to these antibodies and measured the expansion of CD4^+^ and CD8^+^ T cells, as well as the cytotoxic activity of the generated CD8^+^ T cells *in vivo* [227]. White, *et al.* also showed an ovalbumin-specific antibody response after targeting ovalbumin to MHC-II, though it was weaker than utilizing CD11c or CD18 as targeting receptor [208]. Dickgreber, *et al*. and also some other groups tested the usefulness of this receptor for antigen delivery to professional antigen presenting cells, even though it is not a receptor known to be highly endocytic. In contrast to using an antibody specific for MHC-II, they used the modified bacterial superantigen M1 (a modified version of the superantigen streptococcal mitogenic exotoxin Z-2 that binds to MHC class II molecules, but cannot directly stimulate T cells) and coupled it chemically to the model antigen ovalbumin. In addition to the cross-presentation of this antigen by all splenic DC subsets, they could demonstrate a delayed tumor growth of ovalbumin expressing melanoma cells in a therapeutic model [229].

CD11c, also known as ITGAX, is an alpha integrin highly expressed on murine cDCs, which can also be found to lower extends on other cells of the immune system, such as monocytes, macrophages, a subset of B cells, and pDCs. As CD11c has been regarded as a DC-specific receptor for a long time, scientists started to use it for antigen-delivery purposes. In 2000, Wang, *et al.* demonstrated fast antibody responses to polyclonal goat-anti-hamster antibodies complexed to a hamster-anti-CD11c antibody measured by goat-specific antibodies in the serum of intra-dermally vaccinated mice [263]. Later, the group of Glennie used a CD11c-specific antibody chemically coupled to ovalbumin. Targeting an anti CD11c antibody to CD11c positive cells led to the induction of strong CD4^+^ and CD8^+^ T cell responses, with the latter able to efficiently lyse ovalbumin expressing lymphoma cells (EL4) [227]. The final proof of effectiveness was provided by the group of Guo by taking advantage of a single chain F_v_-specific for CD11c and coupled to the breast cancer tumor antigen Her2/neu in combination with the co-stimulatory TLR ligand CpG. They could not only demonstrate the protective and therapeutic properties of this strategy by using Her2/neu expressing tumor cells, but also the delayed onset of tumor growth in a spontaneous mammary carcinoma mouse model [228].

Another molecule used for the delivery of antigens especially to pDCs is Bst-2 (CD317), also known as PDCA-1, tetherin, or HM1.24, which, in the steady-state, is restricted to this cell type within the murine immune system [264]. However, in dependency of the inflammatory stimulus, Bst-2 is downregulated on pDCs but upregulated on other cell types, such as classical DCs and plasma cells [265]. Sapoznikov, *et al.* used a Bst-2-specific F(ab’)2-ova construct to deliver antigens to pDCs *in vivo* and showed an expansion of ovalbumin-specific transgenic CD8^+^ T cells, which was 1000 times less efficient than DEC205-ova targeting to CD8^+^ DCs. Interestingly, they could also show the induction of CD4^+^ transgenic T cell proliferation by lymph node pDCs, but not by splenic DCs [235]. Along with this, Loschko, *et al.* demonstrated—by targeting with Bst-2 specific whole antibodies mutated in their Fc region—that pDCs induced an efficient expansion of antigen-specific CD4^+^ and CD8^+^ T cells. Moreover, immune responses in naïve animals indicated a potent induction of specific antibody and T cell responses, with strong protective capacities demonstrated by challenging with a vaccinia-ova virus and in a s.c. B16F10-ova melanoma model [142].

The Scavenger receptor CD36 is an important receptor for antigen detection and uptake into a variety of phagocytically active cells, such as macrophages and DCs. Further, CD36 was also described to be expressed on a subset of B cells in the murine spleen. Interestingly, it is only present to a higher extend on murine CD8^+^ DCs of the spleen, but not on CD8^−^ DCs, monocytes, pDCs, or skin DCs [233]. In 2008, Tagliani, *et al.* demonstrated that CD36 might be a useful targeting receptor. This was demonstrated by delivery of ovalbumin antigen into murine CD8^+^ DCs by using a single chain F_v_ fusion protein with a dimerizing γ1-CH3 unit. The authors found that targeting of CD36 led to the induction of CD4^+^ and CD8^+^ antigen-specific T cells, but also to induction of cytotoxic CD8^+^ T cells as well as the differentiation of naïve B cells into ovalbumin-specific antibody producing plasma cells [233]. Recently, Pugholm, *et al.* demonstrated that targeting of CD36 led to the secretion of IL-4 and antibody responses without the administration of co-stimulatory agents *in vitro* and *in vivo*. They also reported these responses to be independent of the injection route (s.c./i.v./i.p) [266].

## 6. Conclusions

In the last decades, DCs have been recognized as professional antigen presenting cells indispensable for the control of the immune system. Many murine studies have utilized antibody mediated delivery of antigens to various kinds of endocytic receptors of DCs both *in vitro* and *in vivo*, and have demonstrated select DC subsets to be important for the induction of different immune responses. However, the actual influence of the respective receptor targeted on shaping the immune response still needs to be determined. One major pitfall of some of the antigen targeting studies is the chemical conjugation of antigens to the antibodies, which makes it difficult to calculate the precise antigen load per antibody. Another drawback for the correct interpretation of some of the results is the underestimated influence of the potential Fc receptor mediated binding of the targeting antibodies. Moreover, expression and function of some of the described endocytic receptors in humans and other animal models, the identities of the antigen presenting cell populations as well as their abilities to regulate immune responses is not completely understood yet [11,153,251,267]. Solving the above-mentioned pitfalls will further accelerate the transfer of this technique of a specific induction of immune responses into the human system. This will help to develop strategies of both therapeutic as well as protective treatments of various kinds of human pathologies.

## Figures and Tables

**Table 1:** Overview of important antigen targeting studies.

	Targeted Population	Antibody Type/Coupling	Used Antigen	*In Vivo* Model	Outcome	Ref.
**DEC205**	Murine CD8^+^ DEC205^+^ DCs	Fusion-protein, no FcR-binding	ovalbumin	Transfer of OT-I and OT-II transgenic T cells	Strong cross-presentation by CD8^+^ DCs targeted with a DEC205 antibody due to the expression of MHC-I machinery	[3]
				Transfer of DO11.10 transgenic T cells	TGFβ-dependent induction of FoxP3^+^ T_regs_	[7]
				Immunization of naïve C57Bl/6 mice	Induction of strong CD8^+^ and weaker CD4^+^ T cell responses	[178]
				Immunization of naïve C57Bl/6 mice	*Ex vivo* induced OT-I and OT-II T cell proliferation	[114]
				Transfer of OT-II transgenic T cells into BDCA-2 transgenic C57Bl/6 mice	CD4^+^ T cell proliferation, differentiation, and humoral responses	[137]
				Ova-expressing B16F10 melanoma cells, protective and therapeutic model	Induction of therapeutic and protective anti-tumor immune responses, ova-specific CD4^+^ and CD8^+^ T cell responses in naive C57Bl/6 mice, strong ova-specific mixed IgG1/IgG2a antibody response	[179]
			ovalbumin/ova-NP, circumsporozoite protein (CSP)	Immunization of naïve C57Bl/6, B10.BR, and BALB/c mice; transfer of CSP transgenic T cells	Induction of T helper cell responses, induction of high titers of hapten-specific IgG, stronger antibody response in comparison to immunization with irradiated sporozoites	[180]
			ovalbumin/α-GalCer	Ova-expressing B16F10 and EG7, transfer of transgenic OT-I cells	Delivery of ova and α-GalCer to CD8^+^ DCs induces iNKT cell, CD8^+^ T cell, and protective and therapeutic anti-tumor responses	[181]
			LcrV	Lethal aerosol challenge with *Y. pestis*	Induction of LcrV-specific antibody response, poorer survival in comparison to targeting with DCIR2	[182]
			NS-1 (Dengue virus)	Lethal intracranial challenge with DENV2 NGC	Improved survival by targeting NS-1 via DEC205 to CD8^+^ DCs, stronger T_H_1 response and IFNγ^+^ T cells	[171]
			HIV gag p24 and p41	Intranasal challenge with vaccinia-gag	Strong and broad T cell and antibody response to HIV gag, reduced severity of vaccinia-gag infection	[183]
			HIV gag p24	Immunization of naïve C57Bl/6 mice	Induction of IFNγ^+^ CD4^+^ and CD8^+^ T cell responses	[184]
				Immunization of naïve mice in combination with several adjuvants	Poly(I:C) strongest adjuvant, signaling via IFNAR necessary for activation of DCs and induction of CD8^+^ T cell response	[185]
				Immunization of naïve C57Bl/6 mice	Long-term HIV-specific immunity within the gastrointestinal tract	[186]
			human cartilage proteoglycan (PG)	PG-induced arthritis with transfer of transgenic PG-specific T cells	Targeting PG to DCs induced reduced arthritis score, lower titer of PG-specific IgG1 and IgG2a, and lower proliferation of CD4^+^ T cells	[187]
			stress-inducible 1 protein of *L. major* (LmSTI1a)	Intradermal or subcutaneous challenge with *L. major*	Induction of IFNγ^+^ CD4^+^ T cell responses, improved survival after challenge with *L. major* in BALB/c mice	[188]
			Her2/neu	Protective breast cancer model, injection of NT2.5 tumor cells into FVB/N mice	Induction of IFNγ^+^ CD4^+^ and CD8^+^ T cell responses, protection in a breast cancer tumor model	[189]
			*Trypanosoma cruzi* amastigote surface protein 2 (ASP-2)	Immunization of naïve C57Bl/6 mice	Induction of IFNγ^+^ CD4^+^ T cells, identification of an immunogenic epitope of ASP-2	[190]
			MimA2	Transfer of diabetogenic transgenic AI4 T cells in NOD mice	Induction of tolerance to MimA2 due to deletion of the transferred T cells	[191]
			BDC2.5 mimitope peptide 1040-63/pro-Insulin	Transfer of transgenic BDC2.5 T cells in NOD mice	Induction of T_reg_ cells by delivery of BDC2.5 mimitope via DEC205, delayed onset of diabetes by delivery of pro-insulin to DCs via DEC205	[192]
			LACK (*L. major*)	Transfer of LACK-specific transgenic T cells	Targeting DEC205 induces IFNγ^+^ T_H_1 cells independent of IL-12, but dependent on CD70	[160]
			survivin	Immunization of naïve mice, depletion of T_regs_	Mainly specific T cells against human survivin; depletion of T_regs_ enhances T cell response against survivin	[193]
			IGRP	Immunization of naïve NOD mice	Reduced type-I diabetes	[194]
			PLP(139-151)	Immunization of SJL/J mice after transfer of 5B6 transgenic T cells	Reduced experimental autoimmune encephalomyelitis (EAE)	[195]
			HEL	Transfer of 3A9 transgenic T cells	T cell tolerance in response to antibody alone, memory response after co-injection of αCD40	[8]
		Fusion protein, FcR-binding?	ovalbumin	Transfer of OT-I and OT-II transgenic T cells	Even mature DCs take up antigens via targeting antibodies; induction of OT-I and OT-II T cell activation and proliferation	[196]
		Fusion-protein, single chain Fv	myelin oligodendrocyte glycoprotein (MOG)	MOG-induced experimental allergic encephalomyelitis (EAE)	Induction of protective and therapeutic responses against EAE by targeting MOG to CD8^+^ DCs	[197]
		Fusion-protein, single chain Fv	hNC16A collagen domain	Immunization of naïve C57Bl/6 mice	Reduced graft rejection	[198]
		Single chain Fv	ova/Plasma membrane vesicles of B16F10-ova cells	Vaccination with liposomes or plasma membrane vesicles; i.v. injection of B16F10-ova cells (Lung metastasis model)	Antigen can be delivered in liposomes or plasma membrane vesicles via coupled scFv against DEC205 to DCs; reduction of tumor growth due to tumor-specific T cells	[199]
	Murine DEC205^+^ LN DCs, CD8^+^ DEC205^+^ spleen DCs	Chemically coupled, FcR-binding?	ovalbumin	Transfer of OT-I and OT-II transgenic T cells; challenge with MO4-ova and vaccinia-ova	Induction of IFNγ^+^ memory T cell responses; vaccination induces protection against MO4-ova and vaccinia-ova; *in vivo* targeting protects in contrast to *ex vivo* loaded DCs	[157]
	Murine CD11c^+^ LN DCs	Chemically coupled, FcR-binding?	ovalbumin	Transfer of OT-I transgenic T cells	T cell tolerance in response to antibody alone, memory response after co-injection of αCD40	[200]
	Murine CD11c^+^ DCs	Chemically coupled, FcR-binding?	ovalbumin/TNCB	Delayed-type hypersensitivity and contact hypersensitivity model	Induction of T_regs_, tolerance in DTH and CHS models	[165]
	Murine dermal and LN DCs	Fusion protein, no FcR-binding	ovalbumin	Transfer of target cells in vaccinated mice	αDEC205 targets LCs and dermal DCs; induces cytotoxic CD8^+^ T cell responses independent of Langerin^+^ DCs (Langerin-DTR mice)	[201]
	Murine LCs and dermal DCs	Fusion protein, no FcR-binding	ovalbumin	Naïve C57Bl/6 mice	LCs are targeted by both Langerin and DEC205 antibodies; only DEC205 induces CD4^+^ and CD8^+^ T cell responses	[202]
	DEC205^+^ DCs	Fusion protein, no FcR-binding	HIV gag p24	Immunization of non-human primates	Induction of broad CD4^+^ and CD8^+^ T cell responses against p24 after targeting of DEC205 in combination with poly ICLC	[203]
	Human DCs	Fusion protein, monoclonal humanized antibody	NY-ESO-1	Phase I trial with NY-ESO-1 positive patients	13/45 patients with stabilized disease, 2/45 with tumor regression; no dose-limiting or grade 3 toxicities reported	[85]
	Human moDCs, DEC205^+^ cells in humanized mice	Fusion protein, FcR-binding	EBNA-1	Vaccination of humanized NOG mice	Activation of EBNA-1 specific autologous T cells; protection against EBV-infected B cells; induction of EBNA-1-specific T cells in humanized mice	[204]
	Human moDCs	Fusion protein, FcR-binding	HIV gag p24	-	Stronger IFNγ^+^ CD8^+^ T cell responses by targeting with DEC205 in comparison to DC-SIGN or MMR	[205]
			NY-ESO-1	-	Induction of CD4^+^ and CD8^+^ T cell responses in PBMCs of NY-ESO-1 seropositive breast cancer patients	[206]
		Fusion protein, single chain Fv	MAGE-A3 epitope	-	Induction of proliferation of TCR-transfected CD4^+^ T cells	[207]
**DCIR2**	Murine CD8^−^ DCIR2^+^ DCs	Fusion-protein, no FcR-binding	ovalbumin	Transfer of OT-I and OT-II transgenic T cells	Mainly CD4^+^ T cell response, stronger expression of MHC-II machinery in CD8^−^ DCs	[3]
				Ova-expressing B16F10 melanoma cells, protective and therapeutic model	Induction of therapeutic and protective anti-tumor immune responses, ova-specific CD4^+^ and CD8^+^ T cell responses in naive C57Bl/6 mice, strong ova-specific mixed IgG1/IgG2a antibody response	[179]
				Immunization of naïve C57Bl/6 mice	*Ex vivo* induced OT-I and OT-II T cell proliferation	[114]
				Immunization of naïve C57Bl/6 mice	Induction of mainly CD4^+^ and weaker CD8^+^ T cell responses	[178]
	Murine dermal and LN DCs	Fusion protein, no FcR-binding	LcrV	Lethal aerosol challenge with *Y. pestis*	Induction of LcrV-specific antibody response, improved survival after targeting of DCIR2	[182]
	Murine LCs and dermal DCs	Fusion protein, no FcR-binding	LACK (*L. major*)	Transfer of LACK-specific transgenic T cells	Targeting DCIR2 induces IFNγ^+^ T_H_1 cells dependent on IL-12, but independent of CD70	[160]
	DEC205^+^ DCs	Chemically coupled, Fab-fragment	ovalbumin	Immunization of naïve C57Bl/6 and BALB/c mice	Induction of antibody responses	[208]
	CD34^+^-derived LCs, epidermal LCs, CD11c^+^ blood DCs, blood pDCs	Fusion protein, no FcR-binding	FluMP/MART-1/HIV gag p24	-	Induction of IFNγ^+^ CD8^+^ T cell responses	[174]
	Human blood pDCs	Chemically coupled, FcR-binding?	KLH	-	Proliferation of T cells (not further defined)	[143]
**Clec9a** ****	Murine CD8^+^ DCs	Fusion protein, no FcR-binding	HIV gag p24	Immunization of naïve C57Bl/6 mice	Induction of IFNγ^+^ CD4^+^ and CD8^+^ T cell responses	[184]
		Chemically coupled, FcR-binding?	SIINFEKL/epitopes of gp100, TRP-1, TRP-2	Challenge with B16-ova cells	Protective and therapeutic responses in B16-ova melanoma model	[209]
			ova-peptide (323-339)	Transfer of OT-II cells in C57Bl/6 mice	Differential polarization of naive CD4^+^ T cells dependent on the adjuvant	[210]
			ovalbumin	Immunization of naïve mice	Induction of antibody response without adjuvant and independent of MyD88-signaling; induction of OT-I and OT-II transgenic T cell proliferation	[211]
	Murine CD8^+^ DCs	Fusion protein, single chain Fv	ovalbumin	Transfer of OT-I and OT-II cells	Comparable OT-I T cell activation between DEC205 and Clec9a, superior CD4^+^ T cell responses after targeting of Clec9a	[212]
	Human blood BDCA-3^+^ DCs	Biotin-labeled KLH, gp110-filled nanoparticles, FcR-binding?	KLH/gp100	-	Induction of KLH^+^ CD4^+^ T cell responses, cross-presentation to CD8^+^ T cells	[213]
**Clec12a**	Murine CD8^+^ DCs and pDCs	Chemically coupled, FcR-binding?	ovalbumin	Transfer of OT-I and OT-II cells in C57Bl/6 and CD11c-DTR mice	Induction of OT-I and OT-II responses by CD11c^+^ cells	[214]
**Dectin-1**	Murine DN DCs	Chemically coupled, FcR-binding?	ovalbumin	Immunization of naïve C57Bl/6 mice, transfer of OT-I and OT-II transgenic T cells	Stronger CD4^+^ T cell response and weaker CD8^+^ T cell response in comparison to DEC205-targeting; induction of strong antibody response, especially after *i.v.* injection	[215]
	Human IFNα moDCs	Fusion protein, FcR-binding?	hemagglutinin	-	Re-stimulation of memory T_H_17 cells via antigen-targeting to Dectin-1	[216]
	Human IL-4 or IFNγ DCs	Fusion to hIgG4	Flu M1, MART-1 (26–35)	-	Activation of moDCs by Dectin-1 antibody, expansion of Flu M1 spec & MART-1 CD8^+^ T cells, differentiation of naïve CD8^+^ T cells into Flu M1-specific	[217]
**Langerin**	Murine CD8^+^ DEC205^+^ DCs	Fusion-protein, no FcR-binding	HIV gag p24	Immunization of naïve C57Bl/6 mice	Induction of IFNγ^+^ CD4^+^ and CD8^+^ T cell responses	[184]
	Murine CD11c^+^ Langerin^+^ DCs	Fusion-protein, no FcR-binding	ovalbumin	Immunization of naïve C57Bl/6 mice	Induction of CD4^+^ and CD8^+^ T cell responses	[178]
	Murine LCs and dermal DCs	Fusion-protein, no FcR-binding	ovalbumin	B16-ova model	Targeting of LCs with Langerin and Imiquimod led to cross-tolerance and impaired secondary memory response using DEC205 as targeting antibody	[218]
				Naïve C57Bl/6 mice	LCs are targeted by both Langerin and DEC205 antibodies; only DEC205 induces CD4^+^ and CD8^+^ T cell responses	[202]
	Murine LCs and dermal DCs	Fusion-protein, no FcR-binding	MOGp	Naïve C57Bl/6 mice, partly transfer of MOG-specific T cells	Langerin^+^ cells can induce tolerance *in vivo*	[172]
**DC-SIGN**	Human moDCs	Chemically coupled, FcR-binding?	KLH	-	Proliferation of T cells	[219]
	Murine CD11c^+^ cells expressing hDC-SIGN	Chemically coupled, FcR-binding?	ovalbumin	Infection of humanized mice with *Listeria monocytogenes*	Vaccination with DC-SIGN-ova protects humanized mice from infection with *Listeria monocytogenes*	[220]
				Transfer of OT-I T cells into hDC-SIGN expressing C57Bl/6 mice	Prolonged antigen residence in early endosomes, delayed lysosomal degradation, and cross-presentation	[221]
	DC-SIGN^+^ APCs	-	-	Injection of cynomolgus macaque	APCs in LNs of macaques were targeted	[222]
	Human DC-SIGN^+^ cells	Chemically coupled (KLH), FcR-binding?; fused scFv (tetanus toxoid peptides)	KLH/tetanus toxoid peptides	Immunization of humanized mice	Induction of cell proliferation after targeting of KLH to DC-SIGN^+^ cells; protection after transfer of Raji-cells	[223]
**MR**	Human moDCs	Fusion-protein, FcR-binding?	chorionic gonadotropin β	-	Induction of autologous T cell responses against CGβ; cytotoxic T cells show lysis against CGβ^+^ tumor cell lines (HLA-partially matched)	[224]
			pmel17	-	Induction of autologous T cell responses against pmel17; cytotoxic T cells show lysis against pmel17^+^ melanoma cell lines (HLA-partially matched)	[225]
			chorionic gonadotropin β	-	TLR ligands boosted cytotoxic T cell response induced by antibody targeting	[226]
			NY-ESO-1	-	Antigen targeting to MR induces activation of CD4^+^ and CD8^+^ T cells	[206]
**CD11c**	Murine CD11c^+^ cells	Chemically coupled, Fab-fragment	ovalbumin	Immunization of naïve mice; transferof OT-I and OT-II transgenic T cells; EL4 as target cells	CD11c superior in the generation of CD8^+^ and CD4^+^ T cells; targeting leads to *in vivo* lysis of EL4 cells; induces endogenous ova-specific T cells	[227]
				Immunization of naïve C57Bl/6 and BALB/c mice	Induction of antibody responses	[208]
		Fusion protein, single chain Fv	Her2/neu	Challenge with D2F2/E2 breast cancer cells; spontaneous breast cancer model	Induces protective and therapeutic immune responses against Her2/neu expressing tumor cells; delays tumor growth and onset in a spontaneous breast cancer model	[228]
**MHC-II** ****	MHC-II^+^ APCs	Chemically coupled, Fab-fragment	ovalbumin	Immunization of naïve C57Bl/6 and BALB/c mice	Induction of antibody responses	[208]
	MHC-II^+^ APCs	Chemically coupled, Fab-fragment	ovalbumin	Immunization of naïve C57Bl/6 and BALB/c mice	Induction of antibody responses	[208]
		Chemically coupled, superantigen M1	ovalbumin	*In vivo* killing assays; challenge with B16F10-ova melanoma cells	Cross-presentation by all splenic DC subsets; induces T cell responses with *in vivo* killing activity; therapeutic response in B16F10-ova melanoma model	[229]
**LOX-1**	Not further defined APCs *in vivo*	Chemically coupled, FcR-binding?	ovalbumin	Challenge of C57Bl/6 mice with E.G7 tumor cells expressing ova	Targeting ova to LOX-1 induces protective immune response against E.G7 cells	[230]
	Macaque blood CD11c^+^ and CD14^+^ cells	Fusion protein, FcR-binding?	hemagglutinin	Immunization of rhesus macaques; challenge of macaques with Influenza	Induction of HA-specific antibodies in macaques, higher antibody titer in comparison of Dectin-1 targeting	[231]
	Human IFNα moDCs	Fusion protein, FcR-binding?	hemagglutinin	-	LOX-1 targeting induces activation of T_H_1 cells	[232]
**DC-ASGPR**	Human IFNα moDCs	Fusion protein, FcR-binding?	hemagglutinin	-	DC-ASGPR targeting induces the secretion of IL-10 by DCs and the polarization/re-stimulation of suppressive IL-10^+^ T cells	[232]
**CD36**	Murine CD8^+^ DCs	Fusion protein, scFV	ovalbumin	Challenge of C57Bl/6 mice with E.G7 tumor cells expressing ova	Targeting ova to CD36 induces protective immune response against EG7 tumor cells expressing ova, memory OT-I T cell response without adjuvant	[233]
**Siglec-H**	Murine plasmacytoid DCs	Fusion protein, no FcR-binding	ovalbumin, pHEL, pMOG	EAE	Less severe EAE after targeting of MOG to Siglec-H on pDCs, less T cell polarization, lower antibody titers even after injection of an adjuvant	[141]
			ovalbumin	Transfer of OT-II transgenic T cells into BDCA-2 transgenic C57Bl/6 mice	CD4^+^ T cell proliferation, differentiation, and humoral responses	[137]
	Murine pDCs	Chemically coupled, FcR-binding?	ovalbumin	Immunization of naïve mice, boost with Vaccinia virus expressing ova	Targeting ova via Siglec-H to pDCs induces ova-specific CD8^+^ T cells only when CpG is co-injected	[234]
**BST-2**	Murine pDCs	Fusion protein, no FcR-binding	ovalbumin, pHEL	Vaccinia virus expressing ova, B16F10-ova melanoma cells	Targeting ova to pDCs via BST-2 protects against VV-infection and B16F10-ova cells; induces activation of OT-I and OT-II cells as well as antibody titer	[142]
		Chemically coupled, F(ab)2-fragment	ovalbumin	Transfer of OT-I and OT-II cells	Targeting of ova to pDCs induces OT-II cell proliferation in lymph nodes but not in spleen	[235]
**BDCA-2**	Murine pDCs	Fusion protein, no FcR-binding	ovalbumin	Transfer of OT-II transgenic T cells into BDCA-2 transgenic C57Bl/6 mice	CD4^+^ T cell proliferation, differentiation, and humoral responses	[137]
